# Supplementary material for: A Critical View of the Application of the APEX Software (Aqueous Photochemistry of Environmentally-Occurring Xenobiotics) to Predict Photoreaction Kinetics in Surface Freshwaters
Source: Molecules. 2019 Dec 18;25(1):9. doi: 10.3390/molecules25010009 (PMC7017383; doi:10.3390/molecules25010009)
Supplement: Supplementary file 1 [file molecules-25-00009-s001.zip › 3.2.4_gcc-4.4.0.zip!/3.2.4_gcc-4.4.0/3.2.4_gcc-4.4.0/share/octave/packages/odepkg-0.6.10/doc/odepkg.pdf]

OdePkg 0.6.0

A package for solving ordinary differential equations and more

by Thomas Treichl

Copyright © 2006-2009, Thomas Treichl

Permission is granted to make and distribute verbatim copies of this manual provided the copyright notice and this permission notice are preserved on all copies.

Permission is granted to copy and distribute modified versions of this manual under the conditions for verbatim copying, provided that the entire resulting derived work is distributed under the terms of a permission notice identical to this one.

Permission is granted to copy and distribute translations of this manual into another language, under the same conditions as for modified versions.

Table of Contents

1 Beginners Guide 1

1.1 About OdePkg 1
1.2 OdePkg history and roadmap 1
1.3 Installation and deinstallation 2
1.4 Reporting Bugs 2
1.5 The "foo" example 2

2 Users Guide 5

2.1 Differential Equations 5
2.1.1 ODE equations 5
2.1.2 DAE equations 5
2.1.3 IDE equations 6
2.1.4 DDE equations 6
2.2 Solver families 6
2.2.1 Runge–Kutta solvers 6
2.2.2 Hairer–Wanner solvers 7
2.2.3 Cash modified BDF solvers 8
2.2.4 DDaskr direct method solver 8
2.2.5 Modified Runge–Kutta solvers 8
2.2.6 ODE solver performances 9
2.3 ODE/DAE/IDE/DDE options 10
2.4 M–File Function Reference 17
2.5 Oct–File Function Reference 28

3 Programmers Guide 35

3.1 Missing features 35

Function Index 36

Index 37

1 Beginners Guide

The “Beginners Guide” is intended for users who are new to OdePkg and who want to solve differential equations with the Octave language and the package OdePkg. In this section it will be explained what OdePkg is about in Section 1.1 [About OdePkg], page 1 and how OdePkg grew up from the beginning in Section 1.2 [OdePkg history and roadmap], page 1. In Section 1.3 [Installation and deinstallation], page 2 it is explained how OdePkg can be installed in Octave and how it can later be removed from Octave if it is not needed anymore. If you encounter problems while using OdePkg then have a look at Section 1.4 [Reporting Bugs], page 2 how these bugs can be reported. In the Section 1.5 [The "foo" example], page 2 a first example is explained.

1.1 About OdePkg

OdePkg is part of the **Octave Repository** (resp. the Octave–Forge project) that was initiated by Matthew W. Roberts and Paul Kienzle in the year 2000 and that is hosted at `http://octave.sourceforge.net`. Since then a lot of contributors joined this project and added a lot more packages and functions to further extend the capabilities of GNU Octave.

OdePkg includes commands for setting up various options, output functions etc. before solving a set of differential equations with the solver functions that are included. The package formerly was initiated in autumn 2006 to solve ordinary differential equations (ODEs) only but there are already improvements so that differential algebraic equations (DAEs) in explicit form and in implicit form (IDEs) and delay differential equations (DDEs) can also be solved. The goal of OdePkg is to have a package for solving differential equations that is mostly compatible to proprietary solver products.

1.2 OdePkg history and roadmap

OdePkg Version 0.0.1
: The initial release was a modification of the old “ode” package that is hosted at Octave–Forge and that was written by Marc Compere somewhen between 2000 and 2001. The four variable step–size Runge–Kutta algorithms in three solver files and the three fixed step–size solvers have been merged. It was possible to set some options for these solvers. The four output–functions (`odeprint`, `odeplot`, `odephas2` and `odephas3`) have been added along with other examples that initially have not been there.

OdePkg Version 0.1.x
: The major milestone along versions 0.1.x was that four stable solvers have been implemented (ie. `ode23`, `ode45`, `ode54` and `ode78`) supporting all options that can be set for these kind of solvers and also all necessary functions for setting their options (eg. `odeset`, `odepkg_structure_check, odepkg_event_handle`). Since version 0.1.3 there is also source code available that interfaces the Fortran solver ‘`dopri5.f`’ (that is written by Ernst Hairer and Gerhard Wanner, cf. ‘`odepkg_mexsolver_dopri5.c`’ and the helper files ‘`odepkgext.c`’ and ‘`odepkgmex.c`’).

OdePkg Version 0.2.x
: The main work along version 0.2.x was to make the interface functions for the non–stiff and stiff solvers from Ernst Hairer and Gerhard Wanner enough stable so that they could be compiled and installed by default. Wrapper functions have been added to the package containing a help text and test functions (eg. `ode2r`, `ode5r`, `oders`). Six testsuite functions have been added to check the performance of the different solvers (eg. `odepkg_testsuite_chemakzo`, `odepkg_testsuite_oregonator`).

OdePkg Version 0.3.x — Fixed some minor bugs along version 0.3.x. Thanks to Jeff Cash, who released his Fortran `mebdfX` solvers under the GNU GPL V2 after some discussion. The first IDE solver `odebdi` appeared that is an interface function for Cash's `mebdfi` Fortran core solver. With version 0.3.5 of OdePkg a first new interface function was created based on Octave's C++ `DEFUN_DLD` interface to achieve the highest performance available. Added more examples and testsuite functions (eg. `odepkg_equations_ilorenz`, `odepkg_testsuite_implrober`). Porting all at this time present Mex–file solvers to Octave's C++ `DEFUN_DLD` interface. Ongoing work with this manual.

OdePkg Version 0.4.x — Added a new solver function `odekdi` for the direct method (not the Krylov method) of the '`daskr.f`' solver from the authors Peter N. Brown, Alan C. Hindmarsh, Linda R. Petzold and Clement W. Ulrich that is available under a modified BSD license (without advertising clause). Ongoing work with this manual.

OdePkg Version 0.5.x — Added new solver functions `ode23d`, `ode45d`, `ode54d` and `ode78d` for solving non–stiff delay differential equations (non-stiff DDEs). These solvers are based on the Runge–Kutta solvers `ode23`..`ode78`. Tests and demos have been included for this type of solvers. Added new functions `odeexamples`, `odepkg_examples_ode`, `odepkg_examples_dae`, `odepkg_examples_ide` and `odepkg_examples_ide`. Ongoing work with this manual.

(current) Version 0.6.x — A lot of compatibility tests. Final release before version 1.0.0.

(future) Version 1.0.0 — Completed OdePkg release 1.0.0 with M–solvers and DLD–solvers.

1.3 Installation and deinstallation

OdePkg can be installed easily using the `pkg` command in Octave. To install OdePkg download the latest release of OdePkg from the Octave–Forge download site, then get into that directory where the downloaded release of OdePkg has been saved, start Octave and type

```
pkg install odepkg-x.x.x.tar.gz
```

where '`x.x.x`' in the name of the '`*.tar.gz`' file is the current release number of OdePkg that is available. If you want to deinstall resp. remove OdePkg then simply type

```
pkg uninstall odepkg
```

and make sure that OdePkg has been removed completely and does not appear in the list of installed packages anymore with the following command

```
pkg list
```

1.4 Reporting Bugs

If you encounter problems during the installation process of OdePkg with the `pkg` command or if you have an OdePkg that seems to be broken or if you encounter problems while using OdePkg or if you find bugs in the source codes then please report all of that via email at the Octave–Forge mailing–list using the email address octave-dev@lists.sourceforge.net. Not only bugs are welcome but also any kind of comments are welcome (eg. if you think that OdePkg is absolutely useful or even unnecessary).

1.5 The "foo" example

Have a look at the first ordinary differential equation with the name "`foo`". The `foo` equation of second order may be of the form $y''(t)+C\_1y'(t)+C\_2y(t) = C\_3$. With the substitutions

$y\_1(t) = y(t)$ and $y\_2(t) = y'(t)$ this differential equation of second order can be split into two differential equations of first order, ie. $y'\_1(t) = y\_2(t)$ and $y'\_2(t) = -C\_1 y\_2(t) - C\_2 y\_1(t) + C\_3$. Next the numerical values for the constants need to be defined, ie. $C\_1 = 2.0$, $C\_2 = 5.0$, $C\_3 = 10.0$. This set of ordinary differential equations can then be written as an Octave M–file function like

```
function vdy = foo (vt, vy, varargin)
  vdy(1,1) = vy(2);
  vdy(2,1) = - 2.0 * vy(2) - 5.0 * vy(1) + 10.0;
endfunction
```

It can be seen that this ODEs do not depend on time, nevertheless the first input argument of this function needs to be defined as the time argument *vt* followed by a solution array argument `vy` as the second input argument and a variable size input argument `varargin` that can be used to set up user defined constants or control variables.

As it is known that `foo` is a set of *ordinary* differential equations we can choose one of the four Runge–Kutta solvers (cf. Section 2.2 [Solver families], page 6). It is also known that the time period of interest may be between $t\_0 = 0.0$ and $t\_e = 5.0$ as well as that the initial values of the ODEs are $y\_1(t = 0) = 0.0$ and $y\_2(t = 0) = 0.0$. Solving this set of ODEs can be done by typing the following commands in Octave

```
ode45 (@foo, [0 5], [0 0]);
```

A figure window opens and it can be seen how this ODEs are solved over time. For some of the solvers that come with OdePkg it is possible to define exact time stamps for which an solution is required. Then the example can be called eg.

```
ode45 (@foo, [0:0.1:5], [0 0]);
```

If it is not wanted that a figure window is opened while solving then output arguments have to be used to catch the results of the solving process and to not pass the results to the figure window, eg.

```
[t, y] = ode45 (@foo, [0 5], [0 0]);
```

Results can also be obtained in form of an Octave structure if one output argument is used like in the following example. Then the results are stored in the fields `S.x` and `S.y`.

```
S = ode45 (@foo, [0 5], [0 0]);
```

As noticed before, a function for the ordinary differential equations must not be rewritten all the time if some of the parameters are going to change. That's what the input argument `varargin` can be used for. So rewrite the function `foo` into `newfoo` the following way

```
function vdy = newfoo (vt, vy, varargin)
  vdy(1,1) = vy(2);
  vdy(2,1) = -varargin{1}*vy(2)-varargin{2}*vy(1)+varargin{3};
endfunction
```

There is nothing said anymore about the constant values but if using the following caller routine in the Octave window then the same results can be obtained with the new function `newfoo` as before with the function `foo` (ie. the parameters are directly feed through from the caller routine `ode45` to the function `newfoo`)

```
ode45 (@newfoo, [0 5], [0 0], 2.0, 5.0, 10.0);
```

OdePkg can do much more while solving differential equations, eg. setting up other output functions instead of the function `odeplot` or setting up other tolerances for the solving process etc. As a last example in this beginning chapter it is shown how this can be done, ie. with the command `odeset`

```
A = odeset ('OutputFcn', @odeprint);
ode45 (@newfoo, [0 5], [0 0], A, 2.0, 5.0, 10.0);
```

or

```
A = odeset ('OutputFcn', @odeprint, 'AbsTol', 1e-5, \
            'RelTol', 1e-5, 'NormControl', 'on');
ode45 (@newfoo, [0 5], [0 0], A, 2.0, 5.0, 10.0);
```

The options structure `A` that can be set up with with the command `odeset` must always be the fourth input argument when using the ODE solvers and the DAE solvers but if you are using an IDE solver then `A` must be the fifth input argument (cf. Section 2.2 [Solver families], page 6). The various options that can be set with the command `odeset` are described in Section 2.3 [ODE/DAE/IDE/DDE options], page 10.

Further examples have also been implemented. These example files and functions are of the form `odepkg_examples_*`. Different testsuite examples have been added that are stored in files with filenames `odepkg_testsuite_*`. Before reading the next chapter note that nearly every function that comes with OdePkg has its own help text and its own examples. Look for yourself how the different functions, options and combinations can be used. If you want to have a look at the help description of a special function then type

```
help fcnname
```

in the Octave window where `fcnname` is the name of the function for the help text to be viewed. Type

```
demo fcnname
```

in the Octave window where `fcnname` is the name of the function for the demo to run. Finally write

```
doc odepkg
```

for opening this manual in the texinfo reader of the Octave window.

2 Users Guide

The “Users Guide” is intended for trained users who already know in principal how to solve differential equations with the Octave language and OdePkg. In this chapter it will be explained which equations can be solved with OdePkg in Section 2.1 [Differential Equations], page 5. It will be explained which solvers can be used for the different kind of equations in Section 2.2 [Solver families], page 6 and which options can be set for the optimization of the solving process in Section 2.3 [ODE/DAE/IDE/DDE options], page 10. The help text of all M–file functions and all Oct–file functions have been extracted and are displayed in the sections Section 2.4 [M-File Function Reference], page 17 and Section 2.5 [Oct-File Function Reference], page 28.

2.1 Differential Equations

In this section the different kind of differential equations that can be solved with OdePkg are explained. The formulation of ordinary differential equations is described in section Section 2.1.1 [ODE equations], page 5 followed by the description of explicetly formulated differential algebraic equations in section Section 2.1.2 [DAE equations], page 5, implicetely formulated differential algebraic equations in section Section 2.1.3 [IDE equations], page 6 and delay differential algebraic equations in section Section 2.1.4 [DDE equations], page 6.

2.1.1 ODE equations

ODE equations in general are of the form $y'(t) = f(t, y)$ where $y'(t)$ may be a scalar or vector of derivatives. The variable t always is a scalar describing one point of time and the variable $y(t)$ is a scalar or vector of solutions from the last time step of the set of ordinary differential equations. If the equation is non–stiff then the Section 2.2.1 [Runge-Kutta solvers], page 6 can be used to solve such kind of differential equations but if the equation is stiff then it is recommended to use the Section 2.2.2 [Hairer-Wanner solvers], page 7. An ODE equation definition in Octave must look like

```
function [dy] = ODEequation (t, y, varargin)
```

2.1.2 DAE equations

DAE equations in general are of the form $M(t, y)\backslash cdoty'(t) = f(t, y)$ where $y'(t)$ may be a scalar or vector of derivatives. The variable t always is a scalar describing one point of time and the variable $y(t)$ is a scalar or vector of solutions from the set of differential algebraic equations. The variable $M(t, y)$ is the squared *singular* mass matrix that may depend on y and t. If $M(t, y)$ is not *singular* then the set of equations from above can normally also be written as an ODE equation. If it does not depend on time then it can be defined as a constant matrix or a function. If it does depend on time then it must be defined as a function. Use the command `odeset` to pass the mass matrix information to the solver function (cf. Section 2.3 [ODE/DAE/IDE/DDE options], page 10). If the equation is non–stiff then the Section 2.2.1 [Runge-Kutta solvers], page 6 can be used to solve such kind of differential equations but if the equation is stiff then it is recommended to use the Section 2.2.2 [Hairer-Wanner solvers], page 7. A DAE equation definition in Octave must look like

```
function [dy] = DAEequation (t, y, varargin)
```

and the mass matrix definition can either be a constant mass matrix or a valid function handle to a mass matrix calculation function that can be set with the command `odeset` (cf. option `Mass` of section Section 2.3 [ODE/DAE/IDE/DDE options], page 10).

2.1.3 IDE equations

IDE equations in general are of the form $y'(t)+f(t,y) = 0$ where $y'(t)$ may be a scalar or vector of derivatives. The variable t always is a scalar describing one point of time and the variable $y(t)$ is a scalar or vector of solutions from the set of implicit differential equations. Only IDE solvers from section Section 2.2.3 [Cash modified BDF solvers], page 8 or section Section 2.2.4 [DDaskr direct method solver], page 8 can be used to solve such kind of differential equations. A DAE equation definition in Octave must look like

```
function [residual] = IDEequation (t, y, dy, varargin)
```

2.1.4 DDE equations

DDE equations in general are of the form $y'(t) = f(t, y(t), y(t - \backslash tau\_1), ..., y(t - \backslash tau\_n))$ where $y'(t)$ may be a scalar or vector of derivatives. The variable t always is a scalar describing one point of time and the variables $y(t - \backslash tau\_i)$ are scalars or vectors from the past. Only DDE solvers from section Section 2.2.5 [Modified Runge-Kutta solvers], page 8 can be used to solve such kind of differential equations. A DDE equation definition in Octave must look like

```
function [dy] = DDEequation (t, y, z, varargin)
```

NOTE: Only DDEs with constant delays $y(t - \backslash tau\_i)$ can be solved with OdePkg.

2.2 Solver families

In this section the different kind of solvers are introduced that have been implemented in OdePkg. This section starts with the basic Runge–Kutta solvers in section Section 2.2.1 [Runge-Kutta solvers], page 6 and is continued with the Mex–file Hairer–Wanner solvers in section Section 2.2.2 [Hairer-Wanner solvers], page 7. Performance tests have also been added to the OdePkg. Some of these performance results have been added to section Section 2.2.6 [ODE solver performances], page 9.

2.2.1 Runge–Kutta solvers

The Runge–Kutta solvers are written in the Octave language and that are saved as '`m`'–files. There have been implemented four different solvers with a very similiar structure, ie. `ode23`, `ode45`, `ode54` and `ode78`[1]. The Runge–Kutta solvers have been added to the OdePkg to solve non–stiff ODEs and DAEs, stiff equations of that form cannot be solved with these solvers.

The order of all of the following Runge–Kutta methods is the order of the local truncation error, which is the principle error term in the portion of the Taylor series expansion that gets dropped, or intentionally truncated. This is different from the local error which is the difference between the estimated solution and the actual, or true solution. The local error is used in stepsize selection and may be approximated by the difference between two estimates of different order, $l(h) = x(O(h+1)) - x(O(h))$. With this definition, the local error will be as large as the error in the lower order method. The local truncation error is within the group of terms that gets multipled by h when solving for a solution from the general Runge–Kutta method. Therefore, the order–p solution created by the Runge–Kunge method will be roughly accurate to $O(h\hat{}(p+1))$ since the local truncation error shows up in the solution as $e = h\backslash cdotd$ which is h–times an $O(h\hat{}p)$–term, or rather $O(h\hat{}(p+1))$.

[1] The descriptions for these Runge–Kutta solvers have been taken from the help texts of the initial Runge–Kutta solvers that were written by Marc Compere, he also pointed out that "a relevant discussion on step size choice can be found on page 90ff in U.M. Ascher, L.R. Petzold, Computer Methods for Ordinary Differential Equations and Differential–Agebraic Equations, Society for Industrial and Applied Mathematics (SIAM), Philadelphia, 1998".

`ode23` Integrates a system of non–stiff ordinary differential equations (non–stiff ODEs and DAEs) using second and third order Runge–Kutta formulas. This particular third order method reduces to Simpson's 1/3 rule and uses the third order estimation for the output solutions. Third order accurate Runge–Kutta methods have local and global errors of $O(h\hat{}4)$ and $O(h\hat{}3)$ respectively and yield exact results when the solution is a cubic (the variable h is the step size from one integration step to another integration step). This solver requires three function evaluations per integration step.

`ode45` Integrates a system of non–stiff ordinary differential equations (non–stiff ODEs and DAEs) using fourth and fifth order embedded formulas from Fehlberg. This is a fourth–order accurate integrator therefore the local error normally expected is $O(h\hat{}5)$. However, because this particular implementation uses the fifth–order estimate for x_out (ie. local extrapolation) moving forward with the fifth–order estimate should yield local error of $O(h\hat{}6)$. This solver requires six function evaluations per integration step.

`ode54` Integrates a system of non–stiff ordinary differential equations (non–stiff ODEs and DAEs) using fifth and fourth order Runge–Kutta formulas. The Fehlberg 4(5) of the `ode45` pair is established and works well, however, the Dormand–Prince 5(4) pair minimizes the local truncation error in the fifth–order estimate which is what is used to step forward (local extrapolation). Generally it produces more accurate results and costs roughly the same computationally. This solver requires seven function evaluations per integration step.

`ode78` Integrates a system of non–stiff ordinary differential equations (non–stiff ODEs and DAEs) using seventh and eighth order Runge–Kutta formulas. This is a seventh–order accurate integrator therefore the local error normally expected is $O(h\hat{}8)$. However, because this particular implementation uses the eighth–order estimate for x_out moving forward with the eighth–order estimate will yield errors on the order of $O(h\hat{}9)$. This solver requires thirteen function evaluations per integration step.

2.2.2 Hairer–Wanner solvers

The Hairer–Wanner solvers have been written by Ernst Hairer and Gerhard Wanner. They are written in the Fortran language (hosted at `http://www.unige.ch/~hairer`) and that have been added to the OdePkg as a compressed file with the name '`hairer.tgz`'. Papers and other details about these solvers can be found at the adress given before. The licence of these solvers is a modified BSD license (without advertising clause and therefore are GPL compatible) and can be found as '`licence.txt`' file in the '`hairer.tgz`' package. The Hairer–Wanner solvers have been added to the OdePkg to solve non–stiff and stiff ODEs and DAEs that cannot be solved with any of the Runge–Kutta solvers.

Interface functions for these solvers have been created and have been added to the OdePkg. Their names are '`odepkg_octsolver_XXX.cc`' where '`XXX`' is the name of the Fortran file that is interfaced. The file '`dldsolver.oct`' is created automatically when installing OdePkg with the command `pkg`, but developers can also build each solver manually with the instructions given as a preamble of every '`odepkg_octsolver_XXX.cc`' file.

To provide a short name and to circumvent from the syntax of the original solver function wrapper functions have been added, eg. the command `ode2r` calls the solver `radau` from the Fortran file '`radau.f`'. The other wrapper functions for the Hairer–Wanner solvers are `ode5r` for the `radau5` solver, `oders` for the `rodas` solver and `odesx` for the `seulex` solver. The help text of all these solver functions can be diaplyed by calling `help wrapper` where wrapper is one of `ode2r`, `ode5r`, `oders` or `odesx`.

2.2.3 Cash modified BDF solvers

The backward differentiation algorithm solvers have been written by Jeff Cash in the Fortran language and that are hosted at `http://pitagora.dm.uniba.it/~testset`. They have been added to the OdePkg as a compressed file with the name '`cash.tgz`'. The license of these solvers is a General Public License V2 that can be found as a preamble of each Fortran solver source file. Papers and other details about these solvers can be found at the host adress given before and also at Jeff Cash's homepage at `http://www.ma.ic.ac.uk/~jcash`. Jeff Cash's modified BDF solvers have been added to the OdePkg to solve non–stiff and stiff ODEs and DAEs and also IDEs that cannot be solved with any of the Runge–Kutta solvers.

Interface functions for these solvers have been created and have been added to the OdePkg. Their names are '`odepkg_octsolver_XXX.cc`' where '`XXX`' is the name of the Fortran file that is interfaced. The file '`dldsolver.oct`' is created automatically when installing OdePkg with the command `pkg`, but developers can also build each solver manually with the instructions given as a preamble of every '`odepkg_octsolver_XXX.cc`' file.

To provide a short name and to circumvent from the syntax of the original solver function wrapper functions have been added. The command `odebda` calls the solver `mebdfdae` from the Fortran file '`mebdf.f`' and the `odebdi` calls the solver `mebdfi` from the Fortran file '`mebdfi.f`'.

2.2.4 DDaskr direct method solver

The direct method from the Krylov solver file '`ddaskr.f`' has been written by Peter N. Brown, Alan C. Hindmarsh, Linda R. Petzold and Clement W. Ulrich in the Fortran language and that is hosted at `http://www.netlib.org`. **The Krylov method has not been implemented within OdePkg, only the direct method has been implemented.** The solver and further files for the interface have been added to the OdePkg as a compressed package with the name '`ddaskr.tgz`'. The license of these solvers is a modfied BSD license (without advertising clause) that can be found inside of the compressed package. Other details about this solver can be found as a preamble in the source file '`ddaskr.f`'. The direct method solver of the file '`ddaskr.f`' has been added to the OdePkg to solve non–stiff and stiff IDEs.

An interface function for this solver has been created and has been added to the OdePkg. The source file name is '`odepkg_octsolver_ddaskr.cc`'. The binary function can be found in the file '`dldsolver.oct`' that is created automatically when installing OdePkg with the command `pkg`, but developers can also build the solver wrapper manually with the instructions given as a preamble of the '`odepkg_octsolver_ddaskr.cc`' file.

To provide a short name and to circumvent from the syntax of the original solver function a wrapper function has been added. The command `odekdi` calls the direct method of the solver `ddaskr` from the Fortran file '`ddaskr.f`'.

2.2.5 Modified Runge–Kutta solvers

The modified Runge–Kutta solvers are written in the Octave language and that are saved as m–files. There have been implemented four different solvers that do have a very similiar structure to that solvers found in section Section 2.2.1 [Runge-Kutta solvers], page 6. Their names are `ode23d`, `ode45d`, `ode54d` and `ode78d`. The modified Runge–Kutta solvers have been added to the OdePkg to solve non–stiff DDEs with constant delays only, stiff equations of that form cannot be solved with these solvers. For further information about the error estimation of these solvers cf. section Section 2.2.1 [Runge-Kutta solvers], page 6.

Note: The four DDE solvers of OdePkg are not syntax compatible to propietary solvers. The reason is that the input arguments of the propietary DDE–solvers are completely mixed up in comparison to ODE, DAE and IDE propietary solvers. The DDE solvers of OdePkg have been implemented in form of a syntax compatible way to the other family solvers, eg. propietary solver calls look like

```
ode23  (@fode, vt, vy)         %# for solving an ODE
ode15i (@fide, vt, vy, vdy)    %# for solving an IDE
dde23  (@fdde, vlag, vhist, vt) %# for solving a  DDE
```

whereas in OdePkg the same examples would look like

```
ode23  (@fode, vt, vy)              %# for solving an ODE
odebdi (@fide, vt, vy, vdy)         %# for solving an IDE
ode23d (@fdde, vt, vy, vlag, vhist) %# for solving a  DDE
```

Further, the commands `ddeset` and `ddeget` have not been implemented in OdePkg. Use the functions `odeset` and `odeget` for setting and returning DDE options instead.

2.2.6 ODE solver performances

The following tables give an overview about the performance of the OdePkg ODE/DAE solvers in comparison to propietary solvers when running the HIRES function from the OdePkg testsuite (non–stiff ODE test).

```
>> odepkg ('odepkg_performance_mathires');
--------------------------------------------------------------------------------------------
 Solver  RelTol  AbsTol   Init   Mescd    Scd  Steps  Accept  FEval  JEval  LUdec
--------------------------------------------------------------------------------------------
 ode113  1e-007  1e-007  1e-009   7.57   5.37  24317   21442  45760              11
  ode23  1e-007  1e-007  1e-009   7.23   5.03  13876   13862  41629               2
  ode45  1e-007  1e-007  1e-009   7.91   5.70  11017   10412  66103               2
 ode15s  1e-007  1e-007  1e-009   7.15   4.95    290     273    534      8     59  0
 ode23s  1e-007  1e-007  1e-009   6.24   4.03    702     702   2107    702    702  0
 ode23t  1e-007  1e-007  1e-009   6.00   3.79    892     886   1103      5     72  0
ode23tb  1e-007  1e-007  1e-009   5.85   3.65    735     731   2011      5     66  0
--------------------------------------------------------------------------------------------
```

```
octave:1> odepkg ('odepkg_performance_octavehires');
--------------------------------------------------------------------------------------------
 Solver  RelTol  AbsTol   Init   Mescd    Scd  Steps  Accept  FEval  JEval  LUdec
--------------------------------------------------------------------------------------------
  ode23   1e-07   1e-07  1e-09   7.86   5.44  17112   13369  51333             138
  ode45   1e-07   1e-07  1e-09   8.05   5.63   9397    9393  56376              92
  ode54   1e-07   1e-07  1e-09   8.25   5.83   9300    7758  65093              84
  ode78   1e-07   1e-07  1e-09   8.54   6.12   7290    6615  94757              97
  ode2r   1e-07   1e-07  1e-09   7.69   5.27     50      50    849     50     59  0
  ode5r   1e-07   1e-07  1e-09   7.55   5.13     71      71    671     71     81  0
  oders   1e-07   1e-07  1e-09   7.08   4.66    138     138    828    138    138  0
  odesx   1e-07   1e-07  1e-09   6.56   4.13     30      26   1808     26    205  1
 odebda   1e-07   1e-07  1e-09   6.53   4.11    401     400    582     42     42  0
--------------------------------------------------------------------------------------------
```

The following tables give an overview about the performance of the OdePkg ODE/DAE solvers in comparison to propietary solvers when running the chemical AKZO–NOBEL function from the OdePkg testsuite (non–stiff ODE test).

```
>> odepkg ('odepkg_performance_matchemakzo');
--------------------------------------------------------------------------------------------
 Solver  RelTol  AbsTol   Init   Mescd    Scd  Steps  Accept  FEval  JEval  LUdec
--------------------------------------------------------------------------------------------
 ode113  1e-007  1e-007  1e-007    NaN    Inf      -       -      -      -      -
  ode23  1e-007  1e-007  1e-007    NaN    Inf     15      15     47                0
```

```
   ode45  1e-007  1e-007  1e-007    NaN    Inf     15      15     92
  ode15s  1e-007  1e-007  1e-007   7.04   6.20    161     154              4     35
  ode23s  1e-007  1e-007  1e-007   7.61   6.77   1676    1676   5029   1676   1677
  ode23t  1e-007  1e-007  1e-007   5.95   5.11    406     404              3     39
 ode23tb  1e-007  1e-007  1e-007    NaN    Inf    607            3036      1    608
-------------------------------------------------------------------------------------

octave:1> odepkg ('odepkg_performance_octavechemakzo');
-------------------------------------------------------------------------------------
  Solver  RelTol  AbsTol    Init   Mescd    Scd  Steps  Accept  FEval  JEval  LUdec
-------------------------------------------------------------------------------------
   ode23   1e-07   1e-07   1e-07   2.95   2.06    424     385   1269
   ode45   1e-07   1e-07   1e-07   2.95   2.06    256     218   1530
   ode54   1e-07   1e-07   1e-07   2.95   2.06    197     195   1372
   ode78   1e-07   1e-07   1e-07   2.95   2.06    184     156   2379
   ode2r   1e-07   1e-07   1e-07   8.50   7.57     39      39    372     39     43
   ode5r   1e-07   1e-07   1e-07   8.50   7.57     39      39    372     39     43
   oders   1e-07   1e-07   1e-07   7.92   7.04     67      66    401     66     67
   odesx   1e-07   1e-07   1e-07   7.19   6.26     19      19    457     19     82
  odebda   1e-07   1e-07   1e-07   7.47   6.54    203     203    307     25     25
-------------------------------------------------------------------------------------
```

Other testsuite functions have been added to the OdePkg that can be taken for further performance tests and syntax checks on your own hardware. These functions all have a name '`odepkg_testsuite_XXX.m`' with '`XXX`' being the name of the testsuite equation that has been implemented.

2.3 ODE/DAE/IDE/DDE options

The default values of an OdePkg options structure can be displayed with the command `odeset`. If `odeset` is called without any input argument and one output argument then a OdePkg options structure with default values is created, eg.

```
A = odeset ();
disp (A);
```

There also is an command `odeget` which extracts one or more options from an OdePkg options structure. Other values than default values can also be set with the command `odeset`. The function description of the commands `odeset` and `odeget` can be found in the Section 2.4 [M-File Function Reference], page 17. The values that can be set with the `odeset` command are

'`RelTol`' The option '`RelTol`' is used to set the relative error tolerance for the error estimation of the solver that is used while solving. It can either be a positive scalar or a vector with every element of the vector being a positive scalar (this depends on the solver that is used if both variants are supported). The definite error estimation equation also depends on the solver that is used but generalized (eg. for the solvers `ode23`, `ode45`, `ode54` and `ode78`) it may be a form like $e(t) = max(r\_tol\hat{\ }Ty(t), a\_tol)$. Run the following example to illustrate the effect if this option is used

```
function yd = fvanderpol (vt, vy, varargin)
  mu = 1; ## Set mu > 10 for higher stiffness
  yd = [vy(2); mu * (1 - vy(1)^2) * vy(2) - vy(1)];
endfunction

A = odeset ("RelTol", 1, "OutputFcn", @odeplot);
ode78 (@fvanderpol, [0 20], [2 0], A);
B = odeset (A, "RelTol", 1e-10);
```

```
ode78 (@fvanderpol, [0 20], [2 0], B);
```

'AbsTol' The option 'AbsTol' is used to set the absolute error tolerance for the error estimation of the solver that is used while solving. It can either be a positive scalar or a vector with every element of the vector being a positive scalar (it depends on the solver that is used if both variants are supported). The definite error estimation equation also depends on the solver that is used but generalized (eg. for the solvers ode23, ode45, ode54 and ode78) it may be a form like $e(t) = max(r\_tol\^{}Ty(t), a\_tol)$. Run the following example to illustrate the effect if this option is used

```
## An anonymous implementation of the Van der Pol equation
fvdb = @(vt,vy) [vy(2); (1 - vy(1)^2) * vy(2) - vy(1)];

A = odeset ("AbsTol", 1e-3, "OutputFcn", @odeplot);
ode54 (fvdb, [0 10], [2 0], A);
B = odeset (A, "AbsTol", 1e-10);
ode54 (fvdb, [0 10], [2 0], B);
```

'NormControl'
The option 'NormControl' is used to set the type of error tolerance calculation of the solver that is used while solving. It can either be the string "on" or "off". At the time the solver starts solving a warning message may be displayed if the solver will ignore the "on" setting of this option because of an unhandled resp. missing implementation. If set "on" then the definite error estimation equation also depends on the solver that is used but generalized (eg. for the solvers ode23, ode45, ode54 and ode78) it may be a form like $e(t) = max(r\_tol\^{}Tmax(norm(y(t), \backslash infty)), a\_tol)$. Run the following example to illustrate the effect if this option is used

```
function yd = fvanderpol (vt, vy, varargin)
  mu = 1; ## Set mu > 10 for higher stiffness
  yd = [vy(2); mu * (1 - vy(1)^2) * vy(2) - vy(1)];
endfunction

A = odeset ("NormControl", "on", "OutputFcn", @odeplot);
ode78 (@fvanderpol, [0 20], [2 0], A);
B = odeset (A, "NormControl", "off");
ode78 (@fvanderpol, [0 20], [2 0], B);
```

'MaxStep' The option 'MaxStep' is used to set the maximum step size for the solver that is used while solving. It can only be a positive scalar. By default this value is set internally by every solver and also may differ when using different solvers. Run the following example to illustrate the effect if this option is used

```
function yd = fvanderpol (vt, vy, varargin)
  mu = 1; ## Set mu > 10 for higher stiffness
  yd = [vy(2); mu * (1 - vy(1)^2) * vy(2) - vy(1)];
endfunction

A = odeset ("MaxStep", 10, "OutputFcn", @odeprint);
ode78 (@fvanderpol, [0 20], [2 0], A);
B = odeset (A, "MaxStep", 1e-1);
ode78 (@fvanderpol, [0 20], [2 0], B);
```

'InitialStep'
The option 'InitialStep' is used to set the initial first step size for the solver. It can only be a positive scalar. By default this value is set internally by every solver and also may be different when using different solvers. Run the following example to illustrate the effect if this option is used

```
function yd = fvanderpol (vt, vy, varargin)
  mu = 1; ## Set mu > 10 for higher stiffness
  yd = [vy(2); mu * (1 - vy(1)^2) * vy(2) - vy(1)];
endfunction

A = odeset ("InitialStep", 1, "OutputFcn", @odeprint);
ode78 (@fvanderpol, [0 1], [2 0], A);
B = odeset (A, "InitialStep", 1e-5);
ode78 (@fvanderpol, [0 1], [2 0], B);
```

'`InitialSlope`'
: The option '`InitialSlope`' is not handled by any of the solvers by now.

'`OutputFcn`'
: The option '`OutputFcn`' can be used to set up an output function for displaying the results of the solver while solving. It must be a function handle to a valid function. There are four predefined output functions available with OdePkg. `odeprint` prints the actual time values and results in the Octave window while solving, `odeplot` plots the results over time in a new figure window while solving, `odephas2` plots the first result over the second result as a two–dimensional plot while solving and `odephas3` plots the first result over the second result over the third result as a three–dimensional plot while solving. Run the following example to illustrate the effect if this option is used

```
function yd = fvanderpol (vt, vy, varargin)
  mu = 1; ## Set mu > 10 for higher stiffness
  yd = [vy(2); mu * (1 - vy(1)^2) * vy(2) - vy(1)];
endfunction

A = odeset ("OutputFcn", @odeprint);
ode78 (@fvanderpol, [0 2], [2 0], A);
```

User defined output functions can also be used. A typical framework for a self–made output function may then be of the form

```
function [vret] = odeoutput (vt, vy, vdeci, varargin)
  switch vdeci
    case "init"
      ## Do everything needed to intialize output function
    case "calc"
      ## Do everything needed to create output
    case "done"
      ## Do everything needed to clean up output function
  endswitch
endfunction
```

The output function `odeplot` is also set automatically if the solver calculation routine is called without any output argument. Run the following example to illustrate the effect if this option is not used and no output argument is given

```
## An anonymous implementation of the Van der Pol equation
fvdb = @(vt,vy) [vy(2); (1 - vy(1)^2) * vy(2) - vy(1)];

ode78 (fvdb, [0 20], [2 0]);
```

'`Refine`' The option '`Refine`' is used to set the interpolation factor that is used to increase the quality for the output values if an output function is also set with the option

'OutputFcn'. It can only be a integer value 0< ='Refine'< = 5. Run the following example to illustrate the effect if this option is used

```
function yd = fvanderpol (vt, vy, varargin)
  mu = 1; ## Set mu > 10 for higher stiffness
  yd = [vy(2); mu * (1 - vy(1)^2) * vy(2) - vy(1)];
endfunction

A = odeset ("Refine", 0, "OutputFcn", @odeplot);
ode78 (@fvanderpol, [0 20], [2 0], A);
B = odeset (A, "Refine", 3);
ode78 (@fvanderpol, [0 20], [2 0], B);
```

'OutputSel'

The option 'OutputSel' is used to set the components for which output has to be performed if an output function is also set with the option 'OutputFcn'. It can only be a vector of integer values. Run the following example to illustrate the effect if this option is used

```
function yd = fvanderpol (vt, vy, varargin)
  mu = 1; ## Set mu > 10 for higher stiffness
  yd = [vy(2); mu * (1 - vy(1)^2) * vy(2) - vy(1)];
endfunction

A = odeset ("OutputSel", [1, 2], "OutputFcn", @odeplot);
ode78 (@fvanderpol, [0 20], [2 0], A);
B = odeset (A, "OutputSel", [2]);
ode78 (@fvanderpol, [0 20], [2 0], B);
```

'Stats' The option 'Stats' is used to print cost statistics about the solving process after solving has been finished. It can either be the string "on" or "off". Run the following example to illustrate the effect if this option is used

```
function yd = fvanderpol (vt, vy, varargin)
  mu = 1; ## Set mu > 10 for higher stiffness
  yd = [vy(2); mu * (1 - vy(1)^2) * vy(2) - vy(1)];
endfunction

A = odeset ("Stats", "off");
[a, b] = ode78 (@fvanderpol, [0 2], [2 0], A);
B = odeset ("Stats", "on");
[c, d] = ode78 (@fvanderpol, [0 2], [2 0], B);
```

The cost statistics can also be obtained if the solver calculation routine is called with one output argument. The cost statistics then are in the field 'stats' of the output arguemnt structure. Run the following example to illustrate the effect if this option is used

```
S = ode78 (@fvanderpol, [0 2], [2 0], B);
disp (S);
```

'Jacobian'

The option 'Jacobian' can be used to set up an external Jacobian function or Jacobian matrix for DAE solvers to achieve faster and better results (ODE Runge–Kutta solvers do not need to handle a Jacobian function handle or Jacobian matrix). It must either be a function handle to a valid function or a full constant matrix of size squared the dimension of the set of differential equations. User defined Jacobian

functions must have the form '`function [vjac] = fjac (vt, vy, varargin)`'. Run the following example to illustrate the effect if this option is used

```
function vdy = fpol (vt, vy, varargin)
  vdy = [vy(2); (1 - vy(1)^2) * vy(2) - vy(1)];
endfunction

function vr = fjac (vt, vy, varargin)
  vr = [0, 1; ...
        -1-2*vy(1)*vy(2), 1-vy(1)^2];
endfunction

A = odeset ("Stats", "on");
B = ode5r (@fpol, [0 20], [2 0], A);
C = odeset (A, "Jacobian", @fjac);
D = ode5r (@fpol, [0 20], [2 0], C);
```

Note: The function definition for Jacobian calculations of IDE equations must have the form '`function [vjac, vdjc] = fjac (vt, vy, vyd, varargin)`'. Run the following example to illustrate the effect if this option is used

```
function [vres] = fvanderpol (vt, vy, vyd, varargin)
  vres = [vy(2) - vyd(1);
          (1 - vy(1)^2) * vy(2) - vy(1) - vyd(2)];
endfunction

function [vjac, vdjc] = fjacobian (vt, vy, vyd, varargin)
  vjac = [0, 1; -1 - 2 * vy(1) * vy(2), 1 - vy(1)^2];
  vdjc = [-1, 0; 0, -1];
endfunction

vopt = odeset ("Jacobian", @fjacobian, "Stats", "on");
vsol = odebdi (@fvanderpol, [0, 20], [2; 0], [0; -2], vopt, 10);
```

'`JPattern`'
The option '`JPattern`' is not handled by any of the solvers by now.

'`Vectorized`'
The option '`Vectorized`' is not handled by any of the solvers by now.

'`Mass`' The option '`Mass`' can be used to set up an external Mass function or Mass matrix for solving DAE equations. It depends on the solver that is used if '`Mass`' is supported or not. It must either be a function handle to a valid function or a full constant matrix of size squared the dimension of the set of differential equations. User defined Jacobian functions must have the form '`function vmas = fmas (vt, vy, varargin)`'. Run the following example to illustrate the effect if this option is used

```
function vdy = frob (t, y, varargin)
  vdy(1,1) = -0.04*y(1)+1e4*y(2)*y(3);
  vdy(2,1) =  0.04*y(1)-1e4*y(2)*y(3)-3e7*y(2)^2;
  vdy(3,1) =  y(1)+y(2)+y(3)-1;
endfunction

function vmas = fmas (vt, vy, varargin)
```

```
  vmas =  [1, 0, 0; 0, 1, 0; 0, 0, 0];
endfunction

A = odeset ("Mass", @fmas);
B = ode5r (@frob, [0 1e8], [1 0 0], A);
```

Note: The function definition for Mass calculations of DDE equations must have the form '`function vmas = fmas (vt, vy, vz, varargin)`'.

'`MStateDependence`'

The option '`MStateDependence`' can be used to set up the type of the external Mass function for solving DAE equations if a Mass function handle is set with the option '`Mass`'. It depends on the solver that is used if '`MStateDependence`' is supported or not. It must be a string of the form `"none"`, `"weak"` or `"strong"`. Run the following example to illustrate the effect if this option is used

```
function vdy = frob (vt, vy, varargin)
  vdy(1,1) = -0.04*vy(1)+1e4*vy(2)*vy(3);
  vdy(2,1) =  0.04*vy(1)-1e4*vy(2)*vy(3)-3e7*vy(2)^2;
  vdy(3,1) =  vy(1)+vy(2)+vy(3)-1;
endfunction

function vmas = fmas (vt, varargin)
  vmas =  [1, 0, 0; 0, 1, 0; 0, 0, 0];
endfunction

A = odeset ("Mass", @fmas, "MStateDependence", "none");
B = ode5r (@frob, [0 1e8], [1 0 0], A);
```

User defined Mass functions must have the form as described before (ie. '`function vmas = fmas (vt, varargin)`' if the option '`MStateDependence`' was set to `"none"`, otherwise the user defined Mass function must have the form '`function vmas = fmas (vt, vy, varargin)`' if the option '`MStateDependence`' was set to either `"weak"` or `"strong"`.

'`MvPattern`'

The option '`MvPattern`' is not handled by any of the solvers by now.

'`MassSingular`'

The option '`MassSingular`' is not handled by any of the solvers by now.

'`NonNegative`'

The option '`NonNegative`' can be used to set solution variables to zero even if their real solution would be a negative value. It must be a vector describing the positions in the solution vector for which the option '`NonNegative`' should be used. Run the following example to illustrate the effect if this option is used

```
vfun = @(vt,vy) -abs(vy);
vopt = odeset ("NonNegative", [1]);

[vt1, vy1] = ode78 (vfun, [0 100], [1]);
[vt2, vy2] = ode78 (vfun, [0 100], [1], vopt);

subplot (2,1,1); plot (vt1, vy1);
```

```
subplot (2,1,2); plot (vt2, vy2);
```

'Events' The option 'Events' can be used to set up an Event function, ie. the Event function can be used to find zero crossings in one of the results. It must either be a function handle to a valid function. Run the following example to illustrate the effect if this option is used

```
function vdy = fbal (vt, vy, varargin)
  vdy(1,1) =  vy(2);
  vdy(2,1) = -9.81; ## m/s
endfunction

function [veve, vterm, vdir] = feve (vt, vy, varargin)
  veve  = vy(1); ## Which event component should be tread
  vterm =     1; ## Terminate if an event is found
  vdir  =    -1; ## In which direction, -1 for falling
endfunction

A = odeset ("Events", @feve);
B = ode78 (@fbal, [0 1.5], [1 3], A);
plot (B.x, B.y(:,1));
```

Note: The function definition for Events calculations of DDE equations must have the form '`function [veve, vterm, vdir] = feve (vt, vy, vz, varargin)`' and the function definition for Events calculations of IDE equations must have the form '`function [veve, vterm, vdir] = feve (vt, vy, vyd, varargin)`'.

'MaxOrder'

The option 'MaxOrder' can be used to set the maximum order of the backward differentiation algorithm of the `odebdi` and `odebda` solvers. It must be a scalar integer value between 1 and 7. Run the following example to illustrate the effect if this option is used

```
function res = fwei (t, y, yp, varargin)
  res = t*y^2*yp^3 - y^3*yp^2 + t*yp*(t^2 + 1) - t^2*y;
endfunction

function [dy, dyp] = fjac (t, y, yp, varargin)
  dy  = 2*t*y*yp^3 - 3*y^2*yp^2 - t^2;
  dyp = 3*t*y^2*yp^2 - 2*y^3*yp + t*(t^2 + 1);
endfunction

A = odeset ("AbsTol", 1e-6, "RelTol", 1e-6, "Jacobian", @fjac, ...
            "Stats", "on", "MaxOrder", 1, "BDF", "on")
B = odeset (A, "MaxOrder", 5)
C = odebdi (@fwei, [1 10], 1.2257, 0.8165, A);
D = odebdi (@fwei, [1 10], 1.2257, 0.8165, B);
plot (C.x, C.y, "bo-", D.x, D.y, "rx:");
```

'BDF' The option 'BDF' is only supported by the `odebdi` and `odebda` solvers. Using these solvers the option 'BDF' will automatically be set `"on"` (even if it was set `"off"` before) because the `odebdi` and `odebda` solvers all use the backward differentiation algorithm to solve the different kind of equations.

'NewtonTol'

TODO

'MaxNewtonIterations'
TODO

2.4 M–File Function Reference

The help texts of this section are autogenerated and refer to commands that all can be found in the files '*.m'. All commands that are listed below are loaded automatically everytime you launch Octave.

[] = **ode23** (@*fun*, *slot*, *init*, [*opt*], [*par1*, *par2*, ...]) — Function File
[*sol*] = **ode23** (@*fun*, *slot*, *init*, [*opt*], [*par1*, *par2*, ...]) — Command
[*t*, *y*, [*xe*, *ye*, *ie*]] = **ode23** (@*fun*, *slot*, *init*, [*opt*], [*par1*, *par2*, ...]) — Command

This function file can be used to solve a set of non–stiff ordinary differential equations (non–stiff ODEs) or non–stiff differential algebraic equations (non–stiff DAEs) with the well known explicit Runge–Kutta method of order (2,3).

If this function is called with no return argument then plot the solution over time in a figure window while solving the set of ODEs that are defined in a function and specified by the function handle @*fun*. The second input argument *slot* is a double vector that defines the time slot, *init* is a double vector that defines the initial values of the states, *opt* can optionally be a structure array that keeps the options created with the command `odeset` and *par1*, *par2*, ... can optionally be other input arguments of any type that have to be passed to the function defined by @*fun*.

If this function is called with one return argument then return the solution *sol* of type structure array after solving the set of ODEs. The solution *sol* has the fields *x* of type double column vector for the steps chosen by the solver, *y* of type double column vector for the solutions at each time step of *x*, *solver* of type string for the solver name and optionally the extended time stamp information *xe*, the extended solution information *ye* and the extended index information *ie* all of type double column vector that keep the informations of the event function if an event function handle is set in the option argument *opt*.

If this function is called with more than one return argument then return the time stamps *t*, the solution values *y* and optionally the extended time stamp information *xe*, the extended solution information *ye* and the extended index information *ie* all of type double column vector.

For example, solve an anonymous implementation of the Van der Pol equation

```
fvdb = @(vt,vy) [vy(2); (1 - vy(1)^2) * vy(2) - vy(1)];

vopt = odeset ("RelTol", 1e-3, "AbsTol", 1e-3, \
         "NormControl", "on", "OutputFcn", @odeplot);
ode23 (fvdb, [0 20], [2 0], vopt);
```

[] = **ode23d** (@*fun*, *slot*, *init*, *lags*, *hist*, [*opt*], [*par1*, *par2*, ...]) — Function File
[*sol*] = **ode23d** (@*fun*, *slot*, *init*, *lags*, *hist*, [*opt*], [*par1*, *par2*, ...]) — Command
[*t*, *y*, [*xe*, *ye*, *ie*]] = **ode23d** (@*fun*, *slot*, *init*, *lags*, *hist*, [*opt*], [*par1*, *par2*, ...]) — Command

This function file can be used to solve a set of non–stiff delay differential equations (non–stiff DDEs) with a modified version of the well known explicit Runge–Kutta method of order (2,3).

If this function is called with no return argument then plot the solution over time in a figure window while solving the set of DDEs that are defined in a function and specified by

the function handle @*fun*. The second input argument *slot* is a double vector that defines the time slot, *init* is a double vector that defines the initial values of the states, *lags* is a double vector that describes the lags of time, *hist* is a double matrix and describes the history of the DDEs, *opt* can optionally be a structure array that keeps the options created with the command `odeset` and *par1*, *par2*, ... can optionally be other input arguments of any type that have to be passed to the function defined by @*fun*.

If this function is called with one return argument then return the solution *sol* of type structure array after solving the set of DDEs. The solution *sol* has the fields *x* of type double column vector for the steps chosen by the solver, *y* of type double column vector for the solutions at each time step of *x*, *solver* of type string for the solver name and optionally the extended time stamp information *xe*, the extended solution information *ye* and the extended index information *ie* all of type double column vector that keep the informations of the event function if an event function handle is set in the option argument *opt*.

If this function is called with more than one return argument then return the time stamps *t*, the solution values *y* and optionally the extended time stamp information *xe*, the extended solution information *ye* and the extended index information *ie* all of type double column vector.

For example, solve an anonymous implementation of a chaotic behavior

```
fcao = @(vt, vy, vz) [2 * vz / (1 + vz^9.65) - vy];

vopt = odeset ("NormControl", "on", "RelTol", 1e-3);
vsol = ode23d (fcao, [0, 100], 0.5, 2, 0.5, vopt);

vlag = interp1 (vsol.x, vsol.y, vsol.x - 2);
plot (vsol.y, vlag); legend ("fcao (t,y,z)");
```

[] = **ode45** (@*fun*, *slot*, *init*, [*opt*], [*par1*, *par2*, ...]) — Function File
[*sol*] = **ode45** (@*fun*, *slot*, *init*, [*opt*], [*par1*, *par2*, ...]) — Command
[*t*, *y*, [*xe*, *ye*, *ie*]] = **ode45** (@*fun*, *slot*, *init*, [*opt*], [*par1*, *par2*, ...]) — Command

This function file can be used to solve a set of non–stiff ordinary differential equations (non–stiff ODEs) or non–stiff differential algebraic equations (non–stiff DAEs) with the well known explicit Runge–Kutta method of order (4,5).

If this function is called with no return argument then plot the solution over time in a figure window while solving the set of ODEs that are defined in a function and specified by the function handle @*fun*. The second input argument *slot* is a double vector that defines the time slot, *init* is a double vector that defines the initial values of the states, *opt* can optionally be a structure array that keeps the options created with the command `odeset` and *par1*, *par2*, ... can optionally be other input arguments of any type that have to be passed to the function defined by @*fun*.

If this function is called with one return argument then return the solution *sol* of type structure array after solving the set of ODEs. The solution *sol* has the fields *x* of type double column vector for the steps chosen by the solver, *y* of type double column vector for the solutions at each time step of *x*, *solver* of type string for the solver name and optionally the extended time stamp information *xe*, the extended solution information *ye* and the extended index information *ie* all of type double column vector that keep the informations of the event function if an event function handle is set in the option argument *opt*.

If this function is called with more than one return argument then return the time stamps *t*, the solution values *y* and optionally the extended time stamp information *xe*, the extended solution information *ye* and the extended index information *ie* all of type double column vector.

For example, solve an anonymous implementation of the Van der Pol equation

```
fvdb = @(vt,vy) [vy(2); (1 - vy(1)^2) * vy(2) - vy(1)];

vopt = odeset ("RelTol", 1e-3, "AbsTol", 1e-3, \
         "NormControl", "on", "OutputFcn", @odeplot);
ode45 (fvdb, [0 20], [2 0], vopt);
```

[] = **ode45d** (@*fun*, *slot*, *init*, *lags*, *hist*, [*opt*], [*par1*, *par2*, ...]) Function File
[*sol*] = **ode45d** (@*fun*, *slot*, *init*, *lags*, *hist*, [*opt*], [*par1*, *par2*, ...]) Command
[*t*, *y*, [*xe*, *ye*, *ie*]] = **ode45d** (@*fun*, *slot*, *init*, *lags*, *hist*, [*opt*], [*par1*, *par2*, ...]) Command

This function file can be used to solve a set of non–stiff delay differential equations (non–stiff DDEs) with a modified version of the well known explicit Runge–Kutta method of order (4,5).

If this function is called with no return argument then plot the solution over time in a figure window while solving the set of DDEs that are defined in a function and specified by the function handle @*fun*. The second input argument *slot* is a double vector that defines the time slot, *init* is a double vector that defines the initial values of the states, *lags* is a double vector that describes the lags of time, *hist* is a double matrix and describes the history of the DDEs, *opt* can optionally be a structure array that keeps the options created with the command `odeset` and *par1*, *par2*, ... can optionally be other input arguments of any type that have to be passed to the function defined by @*fun*.

If this function is called with one return argument then return the solution *sol* of type structure array after solving the set of DDEs. The solution *sol* has the fields *x* of type double column vector for the steps chosen by the solver, *y* of type double column vector for the solutions at each time step of *x*, *solver* of type string for the solver name and optionally the extended time stamp information *xe*, the extended solution information *ye* and the extended index information *ie* all of type double column vector that keep the informations of the event function if an event function handle is set in the option argument *opt*.

If this function is called with more than one return argument then return the time stamps *t*, the solution values *y* and optionally the extended time stamp information *xe*, the extended solution information *ye* and the extended index information *ie* all of type double column vector.

For example, solve an anonymous implementation of a chaotic behavior

```
fcao = @(vt, vy, vz) [2 * vz / (1 + vz^9.65) - vy];

vopt = odeset ("NormControl", "on", "RelTol", 1e-4);
vsol = ode45d (fcao, [0, 100], 0.5, 2, 0.5, vopt);

vlag = interp1 (vsol.x, vsol.y, vsol.x - 2);
plot (vsol.y, vlag); legend ("fcao (t,y,z)");
```

[] = **ode54** (@*fun*, *slot*, *init*, [*opt*], [*par1*, *par2*, ...]) Function File
[*sol*] = **ode54** (@*fun*, *slot*, *init*, [*opt*], [*par1*, *par2*, ...]) Command
[*t*, *y*, [*xe*, *ye*, *ie*]] = **ode54** (@*fun*, *slot*, *init*, [*opt*], [*par1*, *par2*, ...]) Command

This function file can be used to solve a set of non–stiff ordinary differential equations (non–stiff ODEs) or non–stiff differential algebraic equations (non–stiff DAEs) with the well known explicit Runge–Kutta method of order (5,4).

If this function is called with no return argument then plot the solution over time in a figure window while solving the set of ODEs that are defined in a function and specified by

the function handle `@`*fun*. The second input argument *slot* is a double vector that defines the time slot, *init* is a double vector that defines the initial values of the states, *opt* can optionally be a structure array that keeps the options created with the command `odeset` and *par1*, *par2*, . . . can optionally be other input arguments of any type that have to be passed to the function defined by `@`*fun*.

If this function is called with one return argument then return the solution *sol* of type structure array after solving the set of ODEs. The solution *sol* has the fields *x* of type double column vector for the steps chosen by the solver, *y* of type double column vector for the solutions at each time step of *x*, *solver* of type string for the solver name and optionally the extended time stamp information *xe*, the extended solution information *ye* and the extended index information *ie* all of type double column vector that keep the informations of the event function if an event function handle is set in the option argument *opt*.

If this function is called with more than one return argument then return the time stamps *t*, the solution values *y* and optionally the extended time stamp information *xe*, the extended solution information *ye* and the extended index information *ie* all of type double column vector.

For example, solve an anonymous implementation of the Van der Pol equation

```
fvdb = @(vt,vy) [vy(2); (1 - vy(1)^2) * vy(2) - vy(1)];

vopt = odeset ("RelTol", 1e-3, "AbsTol", 1e-3, \
         "NormControl", "on", "OutputFcn", @odeplot);
ode54 (fvdb, [0 20], [2 0], vopt);
```

[] = **ode54d** (`@`*fun*, *slot*, *init*, *lags*, *hist*, [*opt*], [*par1*, *par2*, . . .]) — Function File
[*sol*] = **ode54d** (`@`*fun*, *slot*, *init*, *lags*, *hist*, [*opt*], [*par1*, *par2*, . . .]) — Command
[*t*, *y*, [*xe*, *ye*, *ie*]] = **ode54d** (`@`*fun*, *slot*, *init*, *lags*, *hist*, [*opt*], [*par1*, *par2*, . . .]) — Command

This function file can be used to solve a set of non–stiff delay differential equations (non–stiff DDEs) with a modified version of the well known explicit Runge–Kutta method of order (2,3).

If this function is called with no return argument then plot the solution over time in a figure window while solving the set of DDEs that are defined in a function and specified by the function handle `@`*fun*. The second input argument *slot* is a double vector that defines the time slot, *init* is a double vector that defines the initial values of the states, *lags* is a double vector that describes the lags of time, *hist* is a double matrix and describes the history of the DDEs, *opt* can optionally be a structure array that keeps the options created with the command `odeset` and *par1*, *par2*, . . . can optionally be other input arguments of any type that have to be passed to the function defined by `@`*fun*.

If this function is called with one return argument then return the solution *sol* of type structure array after solving the set of DDEs. The solution *sol* has the fields *x* of type double column vector for the steps chosen by the solver, *y* of type double column vector for the solutions at each time step of *x*, *solver* of type string for the solver name and optionally the extended time stamp information *xe*, the extended solution information *ye* and the extended index information *ie* all of type double column vector that keep the informations of the event function if an event function handle is set in the option argument *opt*.

If this function is called with more than one return argument then return the time stamps *t*, the solution values *y* and optionally the extended time stamp information *xe*, the extended solution information *ye* and the extended index information *ie* all of type double column vector.

For example, solve an anonymous implementation of a chaotic behavior

```
fcao = @(vt, vy, vz) [2 * vz / (1 + vz^9.65) - vy];

vopt = odeset ("NormControl", "on", "RelTol", 1e-3);
vsol = ode54d (fcao, [0, 100], 0.5, 2, 0.5, vopt);

vlag = interp1 (vsol.x, vsol.y, vsol.x - 2);
plot (vsol.y, vlag); legend ("fcao (t,y,z)");
```

[] = **ode78** (@*fun*, *slot*, *init*, [*opt*], [*par1*, *par2*, ...]) Function File
[*sol*] = **ode78** (@*fun*, *slot*, *init*, [*opt*], [*par1*, *par2*, ...]) Command
[*t*, *y*, [*xe*, *ye*, *ie*]] = **ode78** (@*fun*, *slot*, *init*, [*opt*], [*par1*, *par2*, ...]) Command

This function file can be used to solve a set of non–stiff ordinary differential equations (non–stiff ODEs) or non–stiff differential algebraic equations (non–stiff DAEs) with the well known explicit Runge–Kutta method of order (7,8).

If this function is called with no return argument then plot the solution over time in a figure window while solving the set of ODEs that are defined in a function and specified by the function handle @*fun*. The second input argument *slot* is a double vector that defines the time slot, *init* is a double vector that defines the initial values of the states, *opt* can optionally be a structure array that keeps the options created with the command `odeset` and *par1*, *par2*, ... can optionally be other input arguments of any type that have to be passed to the function defined by @*fun*.

If this function is called with one return argument then return the solution *sol* of type structure array after solving the set of ODEs. The solution *sol* has the fields *x* of type double column vector for the steps chosen by the solver, *y* of type double column vector for the solutions at each time step of *x*, *solver* of type string for the solver name and optionally the extended time stamp information *xe*, the extended solution information *ye* and the extended index information *ie* all of type double column vector that keep the informations of the event function if an event function handle is set in the option argument *opt*.

If this function is called with more than one return argument then return the time stamps *t*, the solution values *y* and optionally the extended time stamp information *xe*, the extended solution information *ye* and the extended index information *ie* all of type double column vector.

For example, solve an anonymous implementation of the Van der Pol equation

```
fvdb = @(vt,vy) [vy(2); (1 - vy(1)^2) * vy(2) - vy(1)];

vopt = odeset ("RelTol", 1e-3, "AbsTol", 1e-3, \
         "NormControl", "on", "OutputFcn", @odeplot);
ode78 (fvdb, [0 20], [2 0], vopt);
```

[] = **ode78d** (@*fun*, *slot*, *init*, *lags*, *hist*, [*opt*], [*par1*, *par2*, ...]) Function File
[*sol*] = **ode78d** (@*fun*, *slot*, *init*, *lags*, *hist*, [*opt*], [*par1*, *par2*, ...]) Command
[*t*, *y*, [*xe*, *ye*, *ie*]] = **ode78d** (@*fun*, *slot*, *init*, *lags*, *hist*, [*opt*], [*par1*, *par2*, ...]) Command

This function file can be used to solve a set of non–stiff delay differential equations (non–stiff DDEs) with a modified version of the well known explicit Runge–Kutta method of order (7,8).

If this function is called with no return argument then plot the solution over time in a figure window while solving the set of DDEs that are defined in a function and specified by the function handle @*fun*. The second input argument *slot* is a double vector that defines the time slot, *init* is a double vector that defines the initial values of the states, *lags* is

a double vector that describes the lags of time, *hist* is a double matrix and describes the history of the DDEs, *opt* can optionally be a structure array that keeps the options created with the command `odeset` and *par1*, *par2*, . . . can optionally be other input arguments of any type that have to be passed to the function defined by `@`*fun*.

If this function is called with one return argument then return the solution *sol* of type structure array after solving the set of DDEs. The solution *sol* has the fields *x* of type double column vector for the steps chosen by the solver, *y* of type double column vector for the solutions at each time step of *x*, *solver* of type string for the solver name and optionally the extended time stamp information *xe*, the extended solution information *ye* and the extended index information *ie* all of type double column vector that keep the informations of the event function if an event function handle is set in the option argument *opt*.

If this function is called with more than one return argument then return the time stamps *t*, the solution values *y* and optionally the extended time stamp information *xe*, the extended solution information *ye* and the extended index information *ie* all of type double column vector.

For example, solve an anonymous implementation of a chaotic behavior

```
fcao = @(vt, vy, vz) [2 * vz / (1 + vz^9.65) - vy];

vopt = odeset ("NormControl", "on", "RelTol", 1e-3);
vsol = ode78d (fcao, [0, 100], 0.5, 2, 0.5, vopt);

vlag = interp1 (vsol.x, vsol.y, vsol.x - 2);
plot (vsol.y, vlag); legend ("fcao (t,y,z)");
```

[] = **odebwe** (@*fun*, *slot*, *init*, [*opt*], [*par1*, *par2*, . . .]) Function File
[*sol*] = **odebwe** (@*fun*, *slot*, *init*, [*opt*], [*par1*, *par2*, . . .]) Command
[*t*, *y*, [*xe*, *ye*, *ie*]] = **odebwe** (@*fun*, *slot*, *init*, [*opt*], [*par1*, *par2*, . . .]) Command

This function file can be used to solve a set of stiff ordinary differential equations (stiff ODEs) or stiff differential algebraic equations (stiff DAEs) with the Backward Euler method.

If this function is called with no return argument then plot the solution over time in a figure window while solving the set of ODEs that are defined in a function and specified by the function handle `@`*fun*. The second input argument *slot* is a double vector that defines the time slot, *init* is a double vector that defines the initial values of the states, *opt* can optionally be a structure array that keeps the options created with the command `odeset` and *par1*, *par2*, . . . can optionally be other input arguments of any type that have to be passed to the function defined by `@`*fun*.

If this function is called with one return argument then return the solution *sol* of type structure array after solving the set of ODEs. The solution *sol* has the fields *x* of type double column vector for the steps chosen by the solver, *y* of type double column vector for the solutions at each time step of *x*, *solver* of type string for the solver name and optionally the extended time stamp information *xe*, the extended solution information *ye* and the extended index information *ie* all of type double column vector that keep the informations of the event function if an event function handle is set in the option argument *opt*.

If this function is called with more than one return argument then return the time stamps *t*, the solution values *y* and optionally the extended time stamp information *xe*, the extended solution information *ye* and the extended index information *ie* all of type double column vector.

For example, solve an anonymous implementation of the Van der Pol equation

```
fvdb = @(vt,vy) [vy(2); (1 - vy(1)^2) * vy(2) - vy(1)];
vjac = @(vt,vy) [0, 1; -1 - 2 * vy(1) * vy(2), 1 - vy(1)^2];
vopt = odeset ("RelTol", 1e-3, "AbsTol", 1e-3, \
         "NormControl", "on", "OutputFcn", @odeplot, \
         "Jacobian",vjac);
odebwe (fvdb, [0 20], [2 0], vopt);
```

[] = **odeexamples** () Function File

Open the differential equations examples menu and allow the user to select a submenu of ODE, DAE, IDE or DDE examples.

[*value*] = **odeget** (*odestruct*, *option*, [*default*]) Function File
[*values*] = **odeget** (*odestruct*, {*opt1*, *opt2*, ...}, [{*def1*, *def2*, ...}]) Command

If this function is called with two input arguments and the first input argument *odestruct* is of type structure array and the second input argument *option* is of type string then return the option value *value* that is specified by the option name *option* in the OdePkg option structure *odestruct*. Optionally if this function is called with a third input argument then return the default value *default* if *option* is not set in the structure *odestruct*.

If this function is called with two input arguments and the first input argument *odestruct* is of type structure array and the second input argument *option* is of type cell array of strings then return the option values *values* that are specified by the option names *opt1*, *opt2*, ... in the OdePkg option structure *odestruct*. Optionally if this function is called with a third input argument of type cell array then return the default value *def1* if *opt1* is not set in the structure *odestruct*, *def2* if *opt2* is not set in the structure *odestruct*, ...

Run examples with the command

```
demo odeget
```

[*ret*] = **odephas2** (*t*, *y*, *flag*) Function File

Open a new figure window and plot the first result from the variable *y* that is of type double column vector over the second result from the variable *y* while solving. The types and the values of the input parameter *t* and the output parameter *ret* depend on the input value *flag* that is of type string. If *flag* is

‘`"init"`’ then *t* must be a double column vector of length 2 with the first and the last time step and nothing is returned from this function,

‘`""`’ then *t* must be a double scalar specifying the actual time step and the return value is false (resp. value 0) for 'not stop solving',

‘`"done"`’ then *t* must be a double scalar specifying the last time step and nothing is returned from this function.

This function is called by a OdePkg solver function if it was specified in an OdePkg options structure with the `odeset`. This function is an OdePkg internal helper function therefore it should never be necessary that this function is called directly by a user. There is only little error detection implemented in this function file to achieve the highest performance.

For example, solve an anonymous implementation of the "Van der Pol" equation and display the results while solving in a 2D plane

```
fvdb = @(vt,vy) [vy(2); (1 - vy(1)^2) * vy(2) - vy(1)];

vopt = odeset ('OutputFcn', @odephas2, 'RelTol', 1e-6);
vsol = ode45 (fvdb, [0 20], [2 0], vopt);
```

[*ret*] = **odephas3** (*t*, *y*, *flag*) Function File

Open a new figure window and plot the first result from the variable *y* that is of type double column vector over the second and the third result from the variable *y* while solving. The types and the values of the input parameter *t* and the output parameter *ret* depend on the input value *flag* that is of type string. If *flag* is

'`"init"`' then *t* must be a double column vector of length 2 with the first and the last time step and nothing is returned from this function,

'`""`' then *t* must be a double scalar specifying the actual time step and the return value is false (resp. value 0) for 'not stop solving',

'`"done"`' then *t* must be a double scalar specifying the last time step and nothing is returned from this function.

This function is called by a OdePkg solver function if it was specified in an OdePkg options structure with the `odeset`. This function is an OdePkg internal helper function therefore it should never be necessary that this function is called directly by a user. There is only little error detection implemented in this function file to achieve the highest performance.

For example, solve the "Lorenz attractor" and display the results while solving in a 3D plane

```
function vyd = florenz (vt, vx)
  vyd = [10 * (vx(2) - vx(1));
         vx(1) * (28 - vx(3));
         vx(1) * vx(2) - 8/3 * vx(3)];
endfunction

vopt = odeset ('OutputFcn', @odephas3);
vsol = ode23 (@florenz, [0:0.01:7.5], [3 15 1], vopt);
```

[] = **odepkg** () Function File

OdePkg is part of the GNU Octave Repository (the Octave–Forge project). The package includes commands for setting up various options, output functions etc. before solving a set of differential equations with the solver functions that are also included. At this time OdePkg is under development with the main target to make a package that is mostly compatible to proprietary solver products.

If this function is called without any input argument then open the OdePkg tutorial in the Octave window. The tutorial can also be opened with the following command

```
doc odepkg
```

[*sol*] = **odepkg_event_handle** (@*fun*, *time*, *y*, *flag*, [*par1*, *par2*, ...]) Function File

Return the solution of the event function that is specified as the first input argument @*fun* in form of a function handle. The second input argument *time* is of type double scalar and specifies the time of the event evaluation, the third input argument *y* either is of type double column vector (for ODEs and DAEs) and specifies the solutions or is of type cell array (for IDEs and DDEs) and specifies the derivatives or the history values, the third input argument *flag* is of type string and can be of the form

'`"init"`' then initialize internal persistent variables of the function `odepkg_event_handle` and return an empty cell array of size 4,

'`"calc"`' then do the evaluation of the event function and return the solution *sol* as type cell array of size 4,

'"done"' then cleanup internal variables of the function `odepkg_event_handle` and return an empty cell array of size 4.

Optionally if further input arguments *par1*, *par2*, ... of any type are given then pass these parameters through `odepkg_event_handle` to the event function.

This function is an OdePkg internal helper function therefore it should never be necessary that this function is called directly by a user. There is only little error detection implemented in this function file to achieve the highest performance.

[] = **odepkg_examples_dae** () Function File

Open the DAE examples menu and allow the user to select a demo that will be evaluated.

[] = **odepkg_examples_dde** () Function File

Open the DDE examples menu and allow the user to select a demo that will be evaluated.

[] = **odepkg_examples_ide** () Function File

Open the IDE examples menu and allow the user to select a demo that will be evaluated.

[] = **odepkg_examples_ode** () Function File

Open the ODE examples menu and allow the user to select a demo that will be evaluated.

[*newstruct*] = **odepkg_structure_check** (*oldstruct*, ["*solver*"]) Function File

If this function is called with one input argument of type structure array then check the field names and the field values of the OdePkg structure *oldstruct* and return the structure as *newstruct* if no error is found. Optionally if this function is called with a second input argument "*solver*" of type string taht specifies the name of a valid OdePkg solver then a higher level error detection is performed. The function does not modify any of the field names or field values but terminates with an error if an invalid option or value is found.

This function is an OdePkg internal helper function therefore it should never be necessary that this function is called directly by a user. There is only little error detection implemented in this function file to achieve the highest performance.

Run examples with the command

```
demo odepkg_structure_check
```

[*mescd*] = **odepkg_testsuite_calcmescd** (*solution*, *reference*, *abstol*, *reltol*) Function File

If this function is called with four input arguments of type double scalar or column vector then return a normalized value for the minimum number of correct digits *mescd* that is calculated from the solution at the end of an integration interval *solution* and a set of reference values *reference*. The input arguments *abstol* and *reltol* are used to calculate a reference solution that depends on the relative and absolute error tolerances.

Run examples with the command

```
demo odepkg_testsuite_calcmescd
```

[*scd*] = **odepkg_testsuite_calcscd** (*solution*, *reference*, *abstol*, *reltol*) Function File

If this function is called with four input arguments of type double scalar or column vector then return a normalized value for the minimum number of correct digits *scd* that is calculated from the solution at the end of an integration interval *solution* and a set of reference values *reference*. The input arguments *abstol* and *reltol* are unused but present because of compatibility to the function `odepkg_testsuite_calcmescd`.

Run examples with the command

```
demo odepkg_testsuite_calcscd
```

[*solution*] = **odepkg_testsuite_chemakzo** (@*solver*, *reltol*) Function File

If this function is called with two input arguments and the first input argument @*solver* is a function handle describing an OdePkg solver and the second input argument *reltol* is a double scalar describing the relative error tolerance then return a cell array *solution* with performance informations about the chemical AKZO Nobel testsuite of differential algebraic equations after solving (DAE–test).

Run examples with the command

```
demo odepkg_testsuite_chemakzo
```

[*solution*] = **odepkg_testsuite_hires** (@*solver*, *reltol*) Function File

If this function is called with two input arguments and the first input argument @*solver* is a function handle describing an OdePkg solver and the second input argument *reltol* is a double scalar describing the relative error tolerance then return a cell array *solution* with performance informations about the HIRES testsuite of ordinary differential equations after solving (ODE–test).

Run examples with the command

```
demo odepkg_testsuite_hires
```

[*solution*] = **odepkg_testsuite_implakzo** (@*solver*, *reltol*) Function File

If this function is called with two input arguments and the first input argument @*solver* is a function handle describing an OdePkg solver and the second input argument *reltol* is a double scalar describing the relative error tolerance then return a cell array *solution* with performance informations about the chemical AKZO Nobel testsuite of implicit differential algebraic equations after solving (IDE–test).

Run examples with the command

```
demo odepkg_testsuite_implakzo
```

[*solution*] = **odepkg_testsuite_implrober** (@*solver*, *reltol*) Function File

If this function is called with two input arguments and the first input argument @*solver* is a function handle describing an OdePkg solver and the second input argument *reltol* is a double scalar describing the relative error tolerance then return a cell array *solution* with performance informations about the implicit form of the modified ROBERTSON testsuite of implicit differential algebraic equations after solving (IDE–test).

Run examples with the command

```
demo odepkg_testsuite_implrober
```

[*solution*] = **odepkg_testsuite_oregonator** (@*solver*, *reltol*) Function File

If this function is called with two input arguments and the first input argument @*solver* is a function handle describing an OdePkg solver and the second input argument *reltol* is a double scalar describing the relative error tolerance then return a cell array *solution* with performance informations about the OREGONATOR testsuite of ordinary differential equations after solving (ODE–test).

Run examples with the command

```
demo odepkg_testsuite_oregonator
```

[*solution*] = **odepkg_testsuite_pollution** (@*solver*, *reltol*) Function File

If this function is called with two input arguments and the first input argument @*solver* is a function handle describing an OdePkg solver and the second input argument *reltol* is a double scalar describing the relative error tolerance then return the cell array *solution* with performance informations about the POLLUTION testsuite of ordinary differential equations after solving (ODE–test).

Run examples with the command

```
demo odepkg_testsuite_pollution
```

[*solution*] = **odepkg_testsuite_robertson** (@*solver*, *reltol*) Function File

If this function is called with two input arguments and the first input argument @*solver* is a function handle describing an OdePkg solver and the second input argument *reltol* is a double scalar describing the relative error tolerance then return a cell array *solution* with performance informations about the modified ROBERTSON testsuite of differential algebraic equations after solving (DAE–test).

Run examples with the command

```
demo odepkg_testsuite_robertson
```

[*solution*] = **odepkg_testsuite_transistor** (@*solver*, *reltol*) Function File

If this function is called with two input arguments and the first input argument @*solver* is a function handle describing an OdePkg solver and the second input argument *reltol* is a double scalar describing the relative error tolerance then return the cell array *solution* with performance informations about the TRANSISTOR testsuite of differential algebraic equations after solving (DAE–test).

Run examples with the command

```
demo odepkg_testsuite_transistor
```

[*ret*] = **odeplot** (*t*, *y*, *flag*) Function File

Open a new figure window and plot the results from the variable *y* of type column vector over time while solving. The types and the values of the input parameter *t* and the output parameter *ret* depend on the input value *flag* that is of type string. If *flag* is

'`"init"`' then *t* must be a double column vector of length 2 with the first and the last time step and nothing is returned from this function,

'`""`' then *t* must be a double scalar specifying the actual time step and the return value is false (resp. value 0) for 'not stop solving',

'`"done"`' then *t* must be a double scalar specifying the last time step and nothing is returned from this function.

This function is called by a OdePkg solver function if it was specified in an OdePkg options structure with the `odeset`. This function is an OdePkg internal helper function therefore it should never be necessary that this function is called directly by a user. There is only little error detection implemented in this function file to achieve the highest performance.

For example, solve an anonymous implementation of the `"Van der Pol"` equation and display the results while solving

```
fvdb = @(vt,vy) [vy(2); (1 - vy(1)^2) * vy(2) - vy(1)];

vopt = odeset ('OutputFcn', @odeplot, 'RelTol', 1e-6);
vsol = ode45 (fvdb, [0 20], [2 0], vopt);
```

[*ret*] = **odeprint** (*t*, *y*, *flag*) Function File

Display the results of the set of differential equations in the Octave window while solving. The first column of the screen output shows the actual time stamp that is given with the input arguemtn *t*, the following columns show the results from the function evaluation that are given by the column vector *y*. The types and the values of the input parameter *t* and the output parameter *ret* depend on the input value *flag* that is of type string. If *flag* is

'"init"' then *t* must be a double column vector of length 2 with the first and the last time step and nothing is returned from this function,

'""' then *t* must be a double scalar specifying the actual time step and the return value is false (resp. value 0) for 'not stop solving',

'"done"' then *t* must be a double scalar specifying the last time step and nothing is returned from this function.

This function is called by a OdePkg solver function if it was specified in an OdePkg options structure with the `odeset`. This function is an OdePkg internal helper function therefore it should never be necessary that this function is called directly by a user. There is only little error detection implemented in this function file to achieve the highest performance.

For example, solve an anonymous implementation of the "Van der Pol" equation and print the results while solving

```
fvdb = @(vt,vy) [vy(2); (1 - vy(1)^2) * vy(2) - vy(1)];

vopt = odeset ('OutputFcn', @odeprint, 'RelTol', 1e-6);
vsol = ode45 (fvdb, [0 20], [2 0], vopt);
```

[*odestruct*] = **odeset** () Function File
[*odestruct*] = **odeset** ("*field1*", *value1*, "*field2*", *value2*, ...) Command
[*odestruct*] = **odeset** (*oldstruct*, "*field1*", *value1*, "*field2*", *value2*, ...) Command
[*odestruct*] = **odeset** (*oldstruct*, *newstruct*) Command

If this function is called without an input argument then return a new OdePkg options structure array that contains all the necessary fields and sets the values of all fields to default values.

If this function is called with string input arguments "*field1*", "*field2*", ... identifying valid OdePkg options then return a new OdePkg options structure with all necessary fields and set the values of the fields "*field1*", "*field2*", ... to the values *value1*, *value2*, ...

If this function is called with a first input argument *oldstruct* of type structure array then overwrite all values of the options "*field1*", "*field2*", ... of the structure *oldstruct* with new values *value1*, *value2*, ... and return the modified structure array.

If this function is called with two input argumnets *oldstruct* and *newstruct* of type structure array then overwrite all values in the fields from the structure *oldstruct* with new values of the fields from the structure *newstruct*. Empty values of *newstruct* will not overwrite values in *oldstruct*.

For a detailed explanation about valid fields and field values in an OdePkg structure aaray have a look at the '`odepkg.pdf`', Section 'ODE/DAE/IDE/DDE options' or run the command `doc odepkg` to open the tutorial.

Run examples with the command

```
demo odeset
```

2.5 Oct–File Function Reference

The help texts of this section are autogenerated and refer to commands that all can be found in the file '`dldsolver.oct`'. The file '`dldsolver.oct`' is generated automatically if you install OdePkg with the command `pkg`. All commands that are listed below are loaded automatically everytime you launch Octave.

[] = **odebda** (@*fun*, *slot*, *init*, [*opt*], [*par1*, *par2*, ...]) Command
[*sol*] = **odebda** (@*fun*, *slot*, *init*, [*opt*], [*par1*, *par2*, ...]) Command
[*t*, *y*, [*xe*, *ye*, *ie*]] = **odebda** (@*fun*, *slot*, *init*, [*opt*], [*par1*, *par2*, ...]) Command

This function file can be used to solve a set of non–stiff or stiff ordinary differential equations (ODEs) and non–stiff or stiff differential algebraic equations (DAEs). This function file is a wrapper file that uses Jeff Cash's Fortran solver 'mebdfdae.f'.

If this function is called with no return argument then plot the solution over time in a figure window while solving the set of ODEs that are defined in a function and specified by the function handle @*fun*. The second input argument *slot* is a double vector that defines the time slot, *init* is a double vector that defines the initial values of the states, *opt* can optionally be a structure array that keeps the options created with the command odeset and *par1*, *par2*, ... can optionally be other input arguments of any type that have to be passed to the function defined by @*fun*.

If this function is called with one return argument then return the solution *sol* of type structure array after solving the set of ODEs. The solution *sol* has the fields *x* of type double column vector for the steps chosen by the solver, *y* of type double column vector for the solutions at each time step of *x*, *solver* of type string for the solver name and optionally the extended time stamp information *xe*, the extended solution information *ye* and the extended index information *ie* all of type double column vector that keep the informations of the event function if an event function handle is set in the option argument *opt*.

If this function is called with more than one return argument then return the time stamps *t*, the solution values *y* and optionally the extended time stamp information *xe*, the extended solution information *ye* and the extended index information *ie* all of type double column vector.

For example,

```
function y = odepkg_equations_lorenz (t, x)
  y = [10 * (x(2) - x(1));
       x(1) * (28 - x(3));
       x(1) * x(2) - 8/3 * x(3)];
endfunction

vopt = odeset ("InitialStep", 1e-3, "MaxStep", 1e-1, \\
               "OutputFcn", @odephas3, "Refine", 5);
odebda (@odepkg_equations_lorenz, [0, 25], [3 15 1], vopt);
```

[] = **odebdi** (@*fun*, *slot*, *y0*, *dy0*, [*opt*], [*P1*, *P2*, ...]) Command
[*sol*] = **odebdi** (@*fun*, *slot*, *y0*, *dy0*, [*opt*], [*P1*, *P2*, ...]) Command
[*t*, *y*, [*xe*, *ye*, *ie*]] = **odebdi** (@*fun*, *slot*, *y0*, *dy0*, [*opt*], [*P1*, *P2*, ...]) Command

This function file can be used to solve a set of non–stiff and stiff implicit differential equations (IDEs). This function file is a wrapper file that uses Jeff Cash's Fortran solver 'mebdfi.f'.

If this function is called with no return argument then plot the solution over time in a figure window while solving the set of IDEs that are defined in a function and specified by the function handle @*fun*. The second input argument *slot* is a double vector that defines the time slot, *y0* is a double vector that defines the initial values of the states, *dy0* is a double vector that defines the initial values of the derivatives, *opt* can optionally be a structure array that keeps the options created with the command odeset and *par1*, *par2*, ... can optionally be other input arguments of any type that have to be passed to the function defined by @*fun*.

If this function is called with one return argument then return the solution *sol* of type structure array after solving the set of IDEs. The solution *sol* has the fields *x* of type double column vector for the steps chosen by the solver, *y* of type double column vector for the solutions at each time step of *x*, *solver* of type string for the solver name and optionally the extended time stamp information *xe*, the extended solution information *ye* and the extended index information *ie* all of type double column vector that keep the informations of the event function if an event function handle is set in the option argument *opt*.

If this function is called with more than one return argument then return the time stamps *t*, the solution values *y* and optionally the extended time stamp information *xe*, the extended solution information *ye* and the extended index information *ie* all of type double column vector.

For example,

```
function res = odepkg_equations_ilorenz (t, y, yd)
  res = [10 * (y(2) - y(1)) - yd(1);
         y(1) * (28 - y(3)) - yd(2);
         y(1) * y(2) - 8/3 * y(3) - yd(3)];
endfunction

vopt = odeset ("InitialStep", 1e-3, "MaxStep", 1e-1, \\
               "OutputFcn", @odephas3, "Refine", 5);
odebdi (@odepkg_equations_ilorenz, [0, 25], [3 15 1], \\
        [120 81 42.333333], vopt);
```

[] = **odekdi** (@*fun*, *slot*, *y0*, *dy0*, [*opt*], [*P1*, *P2*, ...]) Command
[*sol*] = **odekdi** (@*fun*, *slot*, *y0*, *dy0*, [*opt*], [*P1*, *P2*, ...]) Command
[*t*, *y*, [*xe*, *ye*, *ie*]] = **odekdi** (@*fun*, *slot*, *y0*, *dy0*, [*opt*], [*P1*, *P2*, ...]) Command

This function file can be used to solve a set of non–stiff or stiff implicit differential equations (IDEs). This function file is a wrapper file that uses the direct method (not the Krylov method) of Petzold's, Brown's, Hindmarsh's and Ulrich's Fortran solver '`ddaskr.f`'.

If this function is called with no return argument then plot the solution over time in a figure window while solving the set of IDEs that are defined in a function and specified by the function handle @*fun*. The second input argument *slot* is a double vector that defines the time slot, *y0* is a double vector that defines the initial values of the states, *dy0* is a double vector that defines the initial values of the derivatives, *opt* can optionally be a structure array that keeps the options created with the command `odeset` and *par1*, *par2*, ... can optionally be other input arguments of any type that have to be passed to the function defined by @*fun*.

If this function is called with one return argument then return the solution *sol* of type structure array after solving the set of IDEs. The solution *sol* has the fields *x* of type double column vector for the steps chosen by the solver, *y* of type double column vector for the solutions at each time step of *x*, *solver* of type string for the solver name and optionally the extended time stamp information *xe*, the extended solution information *ye* and the extended index information *ie* all of type double column vector that keep the informations of the event function if an event function handle is set in the option argument *opt*.

If this function is called with more than one return argument then return the time stamps *t*, the solution values *y* and optionally the extended time stamp information *xe*, the extended solution information *ye* and the extended index information *ie* all of type double column vector.

For example,

```
function res = odepkg_equations_ilorenz (t, y, yd)
  res = [10 * (y(2) - y(1)) - yd(1);
         y(1) * (28 - y(3)) - yd(2);
         y(1) * y(2) - 8/3 * y(3) - yd(3)];
endfunction

vopt = odeset ("InitialStep", 1e-3, "MaxStep", 1e-1, \\
               "OutputFcn", @odephas3, "Refine", 5);
odekdi (@odepkg_equations_ilorenz, [0, 25], [3 15 1], \\
        [120 81 42.333333], vopt);
```

[] = **ode2r** (@*fun*, *slot*, *init*, [*opt*], [*par1*, *par2*, ...]) Command
[*sol*] = **ode2r** (@*fun*, *slot*, *init*, [*opt*], [*par1*, *par2*, ...]) Command
[*t*, *y*, [*xe*, *ye*, *ie*]] = **ode2r** (@*fun*, *slot*, *init*, [*opt*], [*par1*, *par2*, ...]) Command

This function file can be used to solve a set of non–stiff or stiff ordinary differential equations (ODEs) and non–stiff or stiff differential algebraic equations (DAEs). This function file is a wrapper to Hairer's and Wanner's Fortran solver '`radau.f`'.

If this function is called with no return argument then plot the solution over time in a figure window while solving the set of ODEs that are defined in a function and specified by the function handle @*fun*. The second input argument *slot* is a double vector that defines the time slot, *init* is a double vector that defines the initial values of the states, *opt* can optionally be a structure array that keeps the options created with the command `odeset` and *par1*, *par2*, ... can optionally be other input arguments of any type that have to be passed to the function defined by @*fun*.

If this function is called with one return argument then return the solution *sol* of type structure array after solving the set of ODEs. The solution *sol* has the fields *x* of type double column vector for the steps chosen by the solver, *y* of type double column vector for the solutions at each time step of *x*, *solver* of type string for the solver name and optionally the extended time stamp information *xe*, the extended solution information *ye* and the extended index information *ie* all of type double column vector that keep the informations of the event function if an event function handle is set in the option argument *opt*.

If this function is called with more than one return argument then return the time stamps *t*, the solution values *y* and optionally the extended time stamp information *xe*, the extended solution information *ye* and the extended index information *ie* all of type double column vector.

For example,

```
function y = odepkg_equations_lorenz (t, x)
  y = [10 * (x(2) - x(1));
       x(1) * (28 - x(3));
       x(1) * x(2) - 8/3 * x(3)];
endfunction

vopt = odeset ("InitialStep", 1e-3, "MaxStep", 1e-1, \\
               "OutputFcn", @odephas3, "Refine", 5);
ode2r (@odepkg_equations_lorenz, [0, 25], [3 15 1], vopt);
```

[] = **ode5r** (@*fun*, *slot*, *init*, [*opt*], [*par1*, *par2*, ...]) Command
[*sol*] = **ode5r** (@*fun*, *slot*, *init*, [*opt*], [*par1*, *par2*, ...]) Command
[*t*, *y*, [*xe*, *ye*, *ie*]] = **ode5r** (@*fun*, *slot*, *init*, [*opt*], [*par1*, *par2*, ...]) Command

This function file can be used to solve a set of non–stiff or stiff ordinary differential equations (ODEs) and non–stiff or stiff differential algebraic equations (DAEs). This function file is a wrapper to Hairer's and Wanner's Fortran solver '`radau5.f`'.

If this function is called with no return argument then plot the solution over time in a figure window while solving the set of ODEs that are defined in a function and specified by the function handle @*fun*. The second input argument *slot* is a double vector that defines the time slot, *init* is a double vector that defines the initial values of the states, *opt* can optionally be a structure array that keeps the options created with the command `odeset` and *par1*, *par2*, ... can optionally be other input arguments of any type that have to be passed to the function defined by @*fun*.

If this function is called with one return argument then return the solution *sol* of type structure array after solving the set of ODEs. The solution *sol* has the fields *x* of type double column vector for the steps chosen by the solver, *y* of type double column vector for the solutions at each time step of *x*, *solver* of type string for the solver name and optionally the extended time stamp information *xe*, the extended solution information *ye* and the extended index information *ie* all of type double column vector that keep the informations of the event function if an event function handle is set in the option argument *opt*.

If this function is called with more than one return argument then return the time stamps *t*, the solution values *y* and optionally the extended time stamp information *xe*, the extended solution information *ye* and the extended index information *ie* all of type double column vector.

For example,

```
function y = odepkg_equations_lorenz (t, x)
  y = [10 * (x(2) - x(1));
       x(1) * (28 - x(3));
       x(1) * x(2) - 8/3 * x(3)];
endfunction

vopt = odeset ("InitialStep", 1e-3, "MaxStep", 1e-1, \\
               "OutputFcn", @odephas3, "Refine", 5);
ode5r (@odepkg_equations_lorenz, [0, 25], [3 15 1], vopt);
```

[] = **oders** (@*fun*, *slot*, *init*, [*opt*], [*par1*, *par2*, ...]) Function File
[*sol*] = **oders** (@*fun*, *slot*, *init*, [*opt*], [*par1*, *par2*, ...]) Command
[*t*, *y*, [*xe*, *ye*, *ie*]] = **oders** (@*fun*, *slot*, *init*, [*opt*], [*par1*, *par2*, ...]) Command

This function file can be used to solve a set of non–stiff or stiff ordinary differential equations (ODEs) and non–stiff or stiff differential algebraic equations (DAEs). This function file is a wrapper to Hairer's and Wanner's Fortran solver '`rodas.f`'.

If this function is called with no return argument then plot the solution over time in a figure window while solving the set of ODEs that are defined in a function and specified by the function handle @*fun*. The second input argument *slot* is a double vector that defines the time slot, *init* is a double vector that defines the initial values of the states, *opt* can optionally be a structure array that keeps the options created with the command `odeset` and *par1*, *par2*, ... can optionally be other input arguments of any type that have to be passed to the function defined by @*fun*.

If this function is called with one return argument then return the solution *sol* of type structure array after solving the set of ODEs. The solution *sol* has the fields *x* of type double column vector for the steps chosen by the solver, *y* of type double column vector

for the solutions at each time step of *x*, *solver* of type string for the solver name and optionally the extended time stamp information *xe*, the extended solution information *ye* and the extended index information *ie* all of type double column vector that keep the informations of the event function if an event function handle is set in the option argument *opt*.

If this function is called with more than one return argument then return the time stamps *t*, the solution values *y* and optionally the extended time stamp information *xe*, the extended solution information *ye* and the extended index information *ie* all of type double column vector.

For example,

```
function y = odepkg_equations_lorenz (t, x)
  y = [10 * (x(2) - x(1));
       x(1) * (28 - x(3));
       x(1) * x(2) - 8/3 * x(3)];
endfunction

vopt = odeset ("InitialStep", 1e-3, "MaxStep", 1e-1, \\
               "OutputFcn", @odephas3, "Refine", 5);
oders (@odepkg_equations_lorenz, [0, 25], [3 15 1], vopt);
```

[] = **odesx** (@*fun*, *slot*, *init*, [*opt*], [*par1*, *par2*, ...]) Command
[*sol*] = **odesx** (@*fun*, *slot*, *init*, [*opt*], [*par1*, *par2*, ...]) Command
[*t*, *y*, [*xe*, *ye*, *ie*]] = **odesx** (@*fun*, *slot*, *init*, [*opt*], [*par1*, *par2*, ...]) Command

This function file can be used to solve a set of stiff or non–stiff ordinary differential equations (ODEs) and non–stiff or stiff differential algebraic equations (DAEs). This function file is a wrapper to Hairer's and Wanner's Fortran solver '`seulex.f`'.

If this function is called with no return argument then plot the solution over time in a figure window while solving the set of ODEs that are defined in a function and specified by the function handle @*fun*. The second input argument *slot* is a double vector that defines the time slot, *init* is a double vector that defines the initial values of the states, *opt* can optionally be a structure array that keeps the options created with the command `odeset` and *par1*, *par2*, ... can optionally be other input arguments of any type that have to be passed to the function defined by @*fun*.

If this function is called with one return argument then return the solution *sol* of type structure array after solving the set of ODEs. The solution *sol* has the fields *x* of type double column vector for the steps chosen by the solver, *y* of type double column vector for the solutions at each time step of *x*, *solver* of type string for the solver name and optionally the extended time stamp information *xe*, the extended solution information *ye* and the extended index information *ie* all of type double column vector that keep the informations of the event function if an event function handle is set in the option argument *opt*.

If this function is called with more than one return argument then return the time stamps *t*, the solution values *y* and optionally the extended time stamp information *xe*, the extended solution information *ye* and the extended index information *ie* all of type double column vector.

For example,

```
function y = odepkg_equations_lorenz (t, x)
  y = [10 * (x(2) - x(1));
       x(1) * (28 - x(3));
       x(1) * x(2) - 8/3 * x(3)];
endfunction
```

```
vopt = odeset ("InitialStep", 1e-3, "MaxStep", 1e-1, \\
               "OutputFcn", @odephas3, "Refine", 5);
odesx (@odepkg_equations_lorenz, [0, 25], [3 15 1], vopt);
```

3 Programmers Guide

3.1 Missing features

If somebody want to help improving OdePkg then please contact the Octave–Forge developer team sending your modifications via the mailing–list octave-dev@lists.sourceforge.net. Here is a TODO–list about missing features:

Partial Derivative equations (PDEs) and Boundary Value Equations (BVPs) cannot be solved with the solvers of the OdePkg. The wish for solving PDEs and BVPs definitely is there (maybe you'd like to create another package and call that package PdePkg, which is just an idea).

Some options that can be set with propietary solver products are not available within OdePkg. Have a look at section Section 2.3 [ODE/DAE/IDE/DDE options], page 10 about which options can be set and which options are not supported and help improving the command `odeset`, `odepkg_structure_check` and the solvers that have to deal with these options.

OdePkg currently is missing the command `decic` which computes an initial constraint for IDEs before solving. The command `deval` also is missing that interpolates the results that can be obtained from the solvers after solving and then plots the results in a new figure. However, instead of using `deval` any of the commands `interpX`.

If you want to include your own solver within OdePkg then either code in '`*.m`' or '`*.cc`'. Solvers in '`*.m`' are preferred. Choose a GPL compatible license for your solver and send your solver file to the mailing list.

Before interfacing other solvers make sure that the core solver file is available under a GPL–compatible license (if you'd like to redistribute your wrapper with OdePkg). There can be found a lot of solver files at `http://www.netlib.org` but most of them are not GPL–compatible. Here is a list about authors and their solvers that do have a GPL compatible license so that their codes can be redistributed with OdePkg:

> Cecilia Magherini and Luigi Brugnano from the University of Bari created the BIMD solver that is available at `http://pitagora.dm.uniba.it/~testset/solvers/bimd.php`. This solver can be used to solve stiff DAE equations. The Fortran77 file has been released under the GNU GPL V2.
>
> Francesca Mazzia and Felice Iavernaro from the University of Bari created the GAMD solver that is available at `http://pitagora.dm.uniba.it/~testset/solvers/gamd.php`. This solver can be used to solve stiff DAE equations. The Fortran90 file has been released under the GNU GPL V2 but for OdePkg a Fortran77 implementation would be preferred.
>
> Ernst Hairer and Gerhard Wanner have been written more solvers that are released under a modified BSD license than have been interfaced by OdePkg. Notable solvers that can be found at `http://www.unige.ch/~hairer/software.html` are explicit Runge-Kutta methods `dopri5` and `dop853` and extrapolation methods `odex` and `odex2` for solving ODEs, `retard` and `radar5` for solving DDEs.
>
> Jeff Cash has released some more Fortran77 solvers for different kinds of differential equation problems than are interfaced by OdePkg, check his website at `http://www.ma.ic.ac.uk/~jcash`.

Function Index

ode23 17
ode23d 17
ode2r 31
ode45 18
ode45d 19
ode54 19
ode54d 20
ode5r 31, 32
ode78 21
ode78d 21
odebda 29
odebdi 29
odebwe 22
odeexamples 23
odeget 23
odekdi 30
odephas2 23
odephas3 24
odepkg 24
odepkg_event_handle 24
odepkg_examples_dae 25
odepkg_examples_dde 25
odepkg_examples_ide 25
odepkg_examples_ode 25
odepkg_structure_check 25
odepkg_testsuite_calcmescd 25
odepkg_testsuite_calcscd 25
odepkg_testsuite_chemakzo 26
odepkg_testsuite_hires 26
odepkg_testsuite_implakzo 26
odepkg_testsuite_implrober 26
odepkg_testsuite_oregonator 26
odepkg_testsuite_pollution 26
odepkg_testsuite_robertson 27
odepkg_testsuite_transistor 27
odeplot 27
odeprint 27
oders 32
odeset 28
odesx 33

Index

A

About OdePkg . . . 1
AbsTol option . . . 11

B

BDF option . . . 16
BDF solver . . . 8
Beginners guide . . . 1
bugs . . . 2

C

Cash modified BDF . . . 8

D

dae equations . . . 5
dae options . . . 10
ddaskr solver . . . 8
dde equations . . . 6
dde options . . . 10
decic . . . 35
deinstallation . . . 2
deval . . . 35
differential equations . . . 5

E

Events option . . . 16

F

foo example . . . 2

H

Hairer–Wanner . . . 7
history . . . 1

I

ide equations . . . 6
ide options . . . 10
InitialSlope option . . . 12
InitialStep option . . . 11
installation . . . 2

J

Jacobian option . . . 13
JPattern option . . . 14

M

m–file reference . . . 17
Mass option . . . 14
MassSingular option . . . 15
MaxNewtonIterations option . . . 17
MaxOrder option . . . 16
MaxStep option . . . 11
missing features . . . 35
MStateDependence option . . . 15
MvPattern option . . . 15

N

NewtonTol option . . . 16
NonNegative option . . . 15
NormControl option . . . 11

O

oct–file reference . . . 28
ode equations . . . 5
ode options . . . 10
OutputFcn option . . . 12
OutputSel option . . . 13

P

performance . . . 9
Programmers guide . . . 35

R

Refine option . . . 12
RelTol option . . . 10
roadmap . . . 1
Runge–Kutta . . . 6
Runge–Kutta modified . . . 8

S

solver families . . . 6
Stats option . . . 13

U

Users guide . . . 5

V

Vectorized option . . . 14
